# Grand challenges: Unlocking peak potential, empowering athletes and coaches to reach their optimal level

**DOI:** 10.3389/fpsyg.2024.1388427

**Published:** 2024-03-19

**Authors:** Miguel-Angel Gomez-Ruano

**Affiliations:** Facultad de Ciencias de la Actividad Física y el Deporte, Universidad Politécnica de Madrid, Madrid, Spain

**Keywords:** sport psychology, peak, athletes, optimal level, coaches

## Introduction

Sport psychology is a well-established field of applied research that delves into various aspects concerning athletes, coaches, parents, and other stakeholders in sport, physical activity, and exercise settings. This discipline encompasses both theoretical and practical dimensions, exploring areas, such as exercise psychology, the social psychology of sport behavior, and the implementation of laboratory and field interventions (Piepiora et al., 2021). Moreover, it embraces a diverse range of sports, physical activities, competition levels, recreational settings, age groups, and gender considerations.

The significance of applied research in sport psychology lies in its multifaceted approach to addressing key issues. These include understanding the psychological dynamics of sport participation, recognizing the mental health benefits of involvement in sport, utilizing observational methods for behavioral analysis, validating questionnaires relevant to sport psychology, studying performance under pressure, managing stress in sports, reevaluating theories within the field, and leveraging artificial intelligence to uncover psychological patterns in sports (Arnold and Fletcher, 2021).

Furthermore, the exploration of new approaches and trends in sport psychology faces several challenges. To address these challenges and pave the way for further research, it is imperative to emphasize the importance of recovery, optimization, and training from a sport psychology perspective (Carson et al., 2020; Gillham and Stone, 2020; Eccles et al., 2022). These considerations will shape the future landscape of sport psychology, facilitating a deeper understanding of athletes' psychological wellbeing and performance enhancement strategies.

## Actions related to grand challenges of optimization, recovery, and training in sport psychology

The sport environment requires coaching staff and professionals working with athletes to allow them to reach their highest level of performance and optimal state to play or compete. At this point, it should be clarified that sport psychology does not deal with improvement in the performance itself, but it plays a great role in bringing out the best in the athlete leading to an adequate “optimal” state or recovering from a previous injury or a decreased physical or performance level. Accordingly, the importance of sport psychology as a tool and way to support and assist coaches and athletes during the training process is vital (Durand-Bush et al., 2023). Based on this rationale, sport psychology research is a key aspect in bridging this gap.

At the first level, where performance is the essential factor to be developed, the different stakeholders need to be better prepared in some aspects:

- Activation and self-control can be useful resources and tools for coaches due to their importance during competitions when organizing training drills, providing feedback and biofeedback, managing team dynamics, or supporting the relationships among players (Ferguson and Hall, 2020).
- Research focusing on the players and athletes can concentrate on how to tolerate pressure, train and perform under stressful scenarios, and attempt to manage the aspects that are under their control (to train in a good way) (Arnold and Fletcher, 2021).
- Emotional states and athletes' wellbeing to pursue optimal performance and recovery (Thompson and Schary, 2021; Taylor et al., 2022).
- Stakeholders from sport institutions, such as managers, fans, or family members who are surrounding and supporting athletes, are part of the context that may affect their optimal states to train and compete. Therefore, the analysis of how to promote an adequate environment and a supportive approach is relevant for sport psychology research (Freeman, 2020; Rouquette et al., 2020).

The training process in sports is the core moment where athletes and coaches should be ready to learn or perform as a way of preparation for further competitions and games. Due to the large variety of contexts of training (formative process, youth stages, competitive level, professional settings, etc.), there is a need to prepare players to learn and train adjusting to the expectancies, and then reaching the learning zone and the effective training zone, where athletes can maximize their potential. Moreover, sport psychology can support the training process for reaching an optimal state or recovering from an injury or a low performance where the key aim is to be ready for the competition or to be selected by the coach (Heidari et al., 2019; Balk and Englert, 2020).

Within the training environment, the relationship between coaches and athletes requires the use of tools, routines, and visualization. This aspect is of vital importance in sport psychology for the exchange of information with athletes and players where the key is concentration, to be focused on the main important aspects of the task or the skill. In addition, training in anticipation and anticipating clues is an excellent and adequate method to improve the quality of decision-making training and concentration routines (Dicks et al., 2019; Williams and Jackson, 2019).

The role of the coach is a determinant aspect where the sport psychology applied to practice is extremely useful for developing inter-personal and intra-personal competencies such as follows:

- The exchange of information with players and the way to communicate during tasks and training drills (e.g., Muir and Munroe-Chandler, 2020).
- How to motivate and promote a positive environment and positive behaviors (Holt et al., 2020).
- How to get the athlete's attention during the training tasks and drills promoting the improvement of the learning process (confidence, resilience, self-regulation, training imagery, etc.) (Wright et al., 2022).
- How to be ready to play the game and apply the drills learned during training (Bonk and Tamminen, 2022).

Finally, these approaches are not only exclusive to regular sport but also to the rapidly growing esports that require the application and adaptation of sport psychology tools and resources to promote optimal states to perform (Watson et al., 2021).

## Future perspectives and opportunities

Based on the above rationale and current approaches, some future perspectives and opportunities for research are provided as follows (among others):

- To investigate *the efficacy of activation and self-control techniques* among coaches in enhancing an athlete's performance during competitions and training sessions.
- To explore *innovative psychological interventions aimed at helping athletes* tolerate pressure, perform under stress, and maintain control over controllable aspects to optimize training effectiveness.
- To examine *the relationship between emotional states, wellbeing, and athletic performance* to develop targeted interventions for promoting optimal performance and facilitating recovery.
- To investigate the *impact of stakeholders, such as managers, fans, and family members, on athletes' optimal training and competitive states*, aiming to identify strategies for creating supportive environments.
- To explore strategies for *optimizing training effectiveness across diverse contexts*, including developmental stages, competitive levels, and professional settings, to maximize athlete's potential.
- To enhance *coach-athlete communication and relationship dynamics* in optimizing performance, motivation, and learning outcomes.
- To examine the *efficacy of anticipation training and concentration routines* in enhancing athletes' decision-making abilities and maintaining focus during training and competition.
- To explore interventions aimed at promoting *positive coaching behaviors and environments* to enhance athletes' motivation, confidence, and resilience.
- To investigate the integration of technology, such as virtual reality or biofeedback devices, in sport psychology interventions.
- To explore the application and adaptation of sport psychology principles and techniques in the rapidly growing field of e-sports.

## Author contributions

M-AG-R: Writing—original draft, Writing—review & editing.

## References

[B1] Arnold R. Fletcher D. (2021). Stress, Well-being, and Performance in Sport. London: Routledge. 10.4324/9780429295874

[B2] Balk Y. A. Englert C. (2020). Recovery self-regulation in sport: Theory, research, and practice. Int. J. Sports Sci. Coach. 15, 273–281. 10.1177/1747954119897528

[B3] Bonk D. Tamminen K. A. (2022). Athletes' perspectives of preparation strategies in open-skill sports. J. Appl. Sport Psychol. 34, 825–845. 10.1080/10413200.2021.1875517

[B4] Carson H. J. Robazza C. Collins D. Toner J. Bertollo M. (2020). “Optimizing performance in sport,” in Advancements in mental skills training, eds. M. Bertollo, E. Filho, and P. C. Terry (London: Routledge). 10.4324/9780429025112-3

[B5] Dicks M. Araújo D. van der Kamp J. (2019). “Perception-action for the study of anticipation and decision making,” in Anticipation and decision making in sport (London: Routledge), 181–199. 10.4324/9781315146270-10

[B6] Durand-Bush N. Baker J. van den Berg F. Richard V. Bloom G. A. (2023). The gold medal profile for sport psychology (GMP-SP). J. Appl. Sport Psychol. 35, 547–570. 10.1080/10413200.2022.2055224

[B7] Eccles D. W. Balk Y. Gretton T. W. Harris N. (2022). “The forgotten session”: Advancing research and practice concerning the psychology of rest in athletes. J. Appl. Sport Psychol. 34, 3–24. 10.1080/10413200.2020.1756526

[B8] Ferguson K. N. Hall C. (2020). Sport biofeedback: exploring implications and limitations of its use. Sport Psychol. 34, 232–241. 10.1123/tsp.2019-0109

[B9] Freeman P. (2020). “Social support in sport,” in Handbook of Sport Psychology 447–463. 10.1002/9781119568124.ch21

[B10] Gillham A. Stone C. (2020). Experiences of peak performance in elite American football. Sport Psychol. 34, 23–34. 10.1123/tsp.2018-0132

[B11] Heidari J. Beckmann J. Bertollo M. Brink M. Kallus K. W. Robazza C.. (2019). Multidimensional monitoring of recovery status and implications for performance. Int. J. Sports Physiol. Perform. 14, 2–8. 10.1123/ijspp.2017-0669 29543069

[B12] Holt N. L. Deal C. J. Pankow K. (2020). “Positive youth development through sport,” in Handbook of Sport Psychology, 429–446. 10.1002/9781119568124.ch20

[B13] Muir I. L. Munroe-Chandler K. J. (2020). Using infographics to promote athletes' mental health: recommendations for sport psychology consultants. J. Sport Psychol. Action 11, 143–164. 10.1080/21520704.2020.1738607

[B14] Piepiora P. Rauk-Kubacka A. Kubacki R. (2021). Sport psychology in the physical culture sciences: a review. Pedagogy Psychol. Sport. 7, 61–75. 10.12775/PPS.2021.07.01.003

[B15] Rouquette O. Y. Knight C. J. Lovett V. E. Heuzé J. P. (2020). Parent-athlete relationships: a central but underexamined consideration within sport psychology. Sport Exer. Psychol. Rev. 16:5 10.53841/bpssepr.2020.16.2.5

[B16] Taylor J. Collins D. Ashford M. (2022). Psychological safety in high-performance sport: contextually applicable? Front. Sports Active Liv. 4:823488. 10.3389/fspor.2022.823488 35615347 PMC9125081

[B17] Thompson B. A. Schary D. P. (2021). Well-being therapy: an approach to increase athlete well-being and performance. J. Sport Psychol. Action. 12, 1–10. 10.1080/21520704.2020.1750516

[B18] Watson M. Abbott C. Pedraza-Ramirez I. (2021). A parallel approach to performance and sport psychology work in esports teams. Int. J. Esports 2:52.

[B19] Williams A. M. Jackson R. C. (2019). Anticipation and Decision Making in Sport. London: Routledge. 10.4324/9781315146270

[B20] Wright D. J. Frank C. Bruton A. M. (2022). Recommendations for combining action observation and motor imagery interventions in sport. J. Sport Psychol. Action. 13, 155–167. 10.1080/21520704.2021.1971810

